# Reward processing deficits arise early in familial frontotemporal dementia

**DOI:** 10.3389/fnins.2024.1491972

**Published:** 2024-11-06

**Authors:** Noah G. Cryns, Emily G. Hardy, Ashlin R. K. Roy, Samir Datta, Andrzej Sokolowski, Virginia E. Sturm, Joel H. Kramer, Adam L. Boxer, Bruce L. Miller, Howard J. Rosen, David C. Perry

**Affiliations:** Department of Neurology, Memory and Aging Center, UCSF Weill Institute for Neurosciences, University of California San Francisco, San Francisco, CA, United States

**Keywords:** dementia, bvFTD, reward, frontotemporal dementia, prodromal, motivation

## Abstract

Reward processing involves evaluation of stimuli to inform what an individual works to pursue or avoid. Patients with behavioral variant frontotemporal dementia (bvFTD) often display reward processing changes, including insensitivity to aversive stimuli. It is unknown how early in the disease course reward changes are detectable. We recruited mutation positive (symptomatic and asymptomatic) and negative members of families with known mutations in progranulin (*GRN*), microtubule-associated protein tau (*MAPT*) and chromosome 9 open reading frame 72 (*C9orf72*). The sample included 4 groups: asymptomatic non-carriers (*n* = 34), asymptomatic carriers [Clinical Dementia Rating (CDR) 0, *n* = 16], mildly symptomatic carriers (CDR 0.5, *n* = 10) and bvFTD (sporadic and genetic, *n* = 45). A series of tasks utilized pleasant, unpleasant, and neutral olfactants to probe reward consumption and effort to obtain reward. A group by valence interaction showed unpleasant scent ratings were more positive in groups with greater disease severity [χ^2^(6) = 87.983, *p* < 0.001]. Mildly symptomatic carriers showed a small difference in ratings of pleasant and unpleasant stimuli, similar to bvFTD. In an effort task, where participants chose to avoid or receive scents, mildly symptomatic carriers and bvFTD chose to smell unpleasant scents more frequently than asymptomatic groups, with mildly symptomatic carriers exceeding bvFTD in their frequency of choosing to smell unpleasant scents. In this same task, motivated effort, determined by rate of button press, determined success at obtaining or avoiding scents. Success rate, calculated based on the number of responses where participants’ button presses exceeded an individual threshold set in a practice trial, differed across groups (*p* = 0.048), driven by mildly symptomatic carriers, who were consistently unsuccessful. There was a group difference in variability in button press rate across trials (*p* = 0.007), driven by mildly symptomatic carriers who showed less varied effort between scents. These findings suggest alterations to reward functioning can be detected early in bvFTD, even before meeting diagnostic criteria. These results may aid in identifying distinctive, initial reward changes in bvFTD that can facilitate early and accurate diagnosis and inspire efforts to identify anatomic underpinnings of early symptomatic changes.

## 1 Introduction

Behavioral variant frontotemporal dementia (bvFTD) is an early age of onset neurodegenerative disease with prominent socioemotional symptoms, including apathy, disinhibition, overeating, lack of sympathy or empathy, and repetitive behaviors. Many of these behavioral symptoms reflect changes in processing of rewards such as money, food, alcohol, or social approval, and there is anatomic overlap in brain regions known to be involved in reward processing and those involved in bvFTD (Perry et al., 2014; Perry and Kramer, 2015; Sturm et al., 2017). Prior studies have shown altered aspects of reward functioning in bvFTD (Perry and Kramer, 2015), including abnormal preference for smaller immediate rewards over larger delayed rewards (Godefroy et al., 2024; Beagle et al., 2020; Manuel et al., 2020; Chiong et al., 2016; Bertoux et al., 2015; Rascovsky et al., 2014; Lebreton et al., 2013), elevated aversion to effort (Le Bouc et al., 2023) and decreased sensitivity to punishment (Carlino et al., 2010). In one such prior study, we demonstrated that individuals with bvFTD perceived unpleasant scents as less aversive than controls (Perry et al., 2017). In an effort to obtain reward task, participants with bvFTD were less motivated to avoid unpleasant scents but showed no difference in their motivation to obtain pleasant scents. These findings showed abnormalities in reward processing in patients whose symptoms had progressed to the point of meeting diagnostic criteria for bvFTD; however, it is unknown how early in the disease course these symptoms manifest.

Accurately diagnosing bvFTD in the early stages can be particularly challenging, as impairment on traditional neuropsychological tests can be minimal, and behavioral or emotional symptoms are often misattributed to major depressive disorder, bipolar disorder, or other psychiatric conditions (Woolley et al., 2011). Early identification of bvFTD is crucial in order to provide accurate prognostication and proper treatment; however, the earliest features of bvFTD are not well established. Though diagnostic delay makes it challenging to recruit patients with early, sporadic bvFTD into research, inherited forms of FTD provide an opportunity to identify clinical features that may develop in the stages of illness even before individuals meet diagnostic criteria.

In presymptomatic mutation carriers, atrophy has been identified as one of the first biomarkers of disease progression (Staffaroni et al., 2022), with regions of early atrophy including areas known to be involved in the processing of reward, including subcortical regions like the striatum, amygdala, and thalamus as well as right frontal cortical areas (Bocchetta et al., 2021; Borrego-Écija et al., 2021; Le Blanc et al., 2020; Seeley et al., 2008). Given the anatomical overlap between these earliest regions of atrophy in bvFTD and the reward network, reward deficits may be some of the earliest behavioral markers of disease progression.

Prior literature has identified early behavioral shifts in FTD-related mutation carriers before conversion to bvFTD, including apathy, decreased empathy, loss of socioemotional sensitivity and changes in anxiety and depression (Malpetti et al., 2021; Nelson et al., 2022; Foster et al., 2022; Franklin et al., 2021; Benussi et al., 2021; Toller et al., 2023), though changes in reward-related behavior at this stage are not well explored. In this study, we investigated early reward processing changes in members of families with known FTD-causing mutations, with participants included regardless of individual mutation status, resulting in a sample of mutation negative and mutation positive family members, some presenting when they were asymptomatic and others presenting with mild symptoms. We aimed to study these mutation carriers using reward tasks which have previously shown differences in bvFTD relative to healthy controls, and which are designed to capture distinct elements of reward processing, including consumption and effort to obtain reward.

## 2 Materials and methods

### 2.1 Participants

Patients with behavioral variant frontotemporal dementia (bvFTD) and preclinical (asymptomatic or mildly symptomatic) members of families with known mutations causing frontotemporal dementia [progranulin (*GRN*), microtubule-associated protein tau (*MAPT*) and chromosome 9 open reading frame 72 (*C9orf72*)] were recruited for this study (Table 1). Family members were recruited without awareness of their genetic mutation status. All participants underwent a multidisciplinary evaluation at the University of California San Francisco Memory and Aging Center to arrive at a consensus diagnosis. The evaluation included a battery of neuropsychological tests, an assessment of functional status, and behavioral measures, including the Neuropsychiatric Inventory (NPI). All participants with bvFTD met diagnostic criteria for at least possible bvFTD (Rascovsky et al., 2011). Patients with bvFTD having a score greater than 2 on the Clinical Dementia Rating scale (CDR) (Morris, 1993) were excluded due to concerns that they could not properly complete the task. The bvFTD sample (*n* = 45) consisted of 13 *C9orf72* repeat expansion carriers, 4 *GRN* mutation carriers, 2 *MAPT* mutation carriers, 24 sporadic cases, and 2 without available genetic information. Preclinical members of families with known mutations causing frontotemporal dementia were divided into three groups based on genetic status and impairment: (1) Non-carrier: Non-carriers of the pathogenic mutations with a CDR of 0 (*n* = 34), (2) Asymptomatic carrier: Asymptomatic carriers of pathogenic mutations with a CDR of 0 (*n* = 16: 7 *C9orf72*, 7 *GRN*, and 2 *MAPT*), and (3) Mildly symptomatic carrier: Mildly symptomatic carriers of pathogenic mutations with a CDR of 0.5 (*n* = 10: 6 *C9orf72* and 4 *GRN*). Written consent was obtained from all participants or surrogates in accordance with procedures defined by the UCSF Human Research Protection Program.

**Table 1:** Group demographics and clinical characteristics.

	Non-carriers, *n* = 34	Asymptomatic carriers, *n* = 16	Mildly symptomatic carriers, *n* = 10	bvFTD, *n* = 45	Statistical comparison
Gender (male/female)	16/18	6/10	7/3	27/18	X^2^(3, *n* = 105) = 4.07, *p* = 0.25
Age	46.7 (11.4)^d^	46 (14.1)^d^	51.9 (6.68)^d^	63.8 (7.01)^abc^	*F*(3, 101, *n* = 105) = 24.52, *p* < 0.001
MMSE	28.8 (0.95)^d^	28.6 (1.22)^d^	27 (1.89)^d^	24 (3.43)^abc^	*F*(3, 95, *n* = 99) = 28.46, *p* < 0.001
CDR	0 (0)^cd^	0 (0)^cd^	0.5 (0)^abd^	1.18 (0.57)^abc^	*F*(3, 101, *n* = 105) = 78.64, *p* < 0.001
NPI apathy	0.067 (0.37)^cd^	0.083 (0.29)^cd^	4.56 (3.21)^abd^	8.07 (3.48)^abc^	*F*(3, 87, *n* = 91) = 68.13, *p* < 0.001
NPI depression	0.057 (1.57)	0.17 (0.39)	2.33 (2.06)	1.61 (2.48)	*F*(3, 88, *n* = 92) = 3.58, *p* = 0.017
NPI eating	0.033 (0.18)^d^	0 (0)^d^	1.78 (2.73)^d^	7.2 (3.79)^abc^	*F*(3, 87, *n* = 91) = 50.43, *p* < 0.001
NPI euphoria	0 (0)^d^	0 (0)^d^	0 (0)^d^	4.39 (3.87)^abc^	*F*(3, 88, *n* = 92) = 21.43, *p* < 0.001
NPI disinhibition	0 (0)^d^	0.083 (0.29)^d^	1.44 (2.6)^d^	7.02 (3.32)^abc^	*F*(3, 87, *n* = 91) = 62.09, *p* < 0.001
NPI aberrant motor	0 (0)^d^	0 (0)^d^	0.56 (1.13)^d^	6.62 (3.81)^abc^	*F*(3, 87, *n* = 91) = 48.23, *p* < 0.001

Results displayed as mean (standard deviation) for age, MMSE, CDR, and NPI. MMSE – Mini-Mental State Examination, CDR – Clinical Dementia Rating Scale. NPI – Neuropsychiatric Inventory Questionnaire. Tukey-corrected *post hoc* tests for pairwise comparisons: ^a^different from non-carriers, ^b^different from asymptomatic carriers, ^c^different from mildly symptomatic carriers, and ^d^different from bvFTD at *p* < 0.05.

### 2.2 Procedure

Participants were seated in front of a computer screen and completed a battery of tasks relevant to reward processing, including a reward consumption task, an effort to obtain reward task, and an odor discrimination task. In the reward consumption and effort tasks, participants were administered a series of seven olfactants. They were instructed to inhale for 3 s, exhale for 3 s, then on the next inhalation, a glass vial containing one of the 7 olfactants was held beneath their nose, and they were instructed to sniff. Each task involved 3 pleasant olfactants, 3 unpleasant olfactants, and one neutral olfactant, all diluted in propylene glycol. The pleasant valence olfactants were 8% vanillin, 10% menthol, and 10% citral. Unpleasant olfactants included 5% isovaleric acid, 1% propionic acid, and 1% pyridine. 100% propylene glycol was used for the neutral olfactant. All chemical olfactants were obtained from Sigma-Aldrich and have previously been found to have the desired valence in healthy controls (Alaoui-Ismaïli et al., 1997; Bensafi et al., 2002; Anderson et al., 2003; Rolls et al., 2003). E-prime was used to administer the tasks. Eighty-six participants were tested using E-prime 2.0, and 19 participants were tested using E-prime 3.0.

#### 2.2.1 Reward consumption task

In keeping with terminology previously used to describe the stage of reward processing in which a rewarding stimulus is received, we refer to this initial test as the “reward consumption” task (Diez et al., 2024; Chan and Chou, 2022; Korb et al., 2020; Rademacher et al., 2010). The 7 olfactants were administered to participants in random order. After sniffing each one, participants were asked to choose a single number between 1 and 9 reflecting how pleasant they perceived each scent; with 1 being very unpleasant, 9 being extremely pleasant and 5 being neutral. Stimuli were delivered only once. Intervals between scents were variable depending on how long each participant took to respond. The minimum interval between scent delivery was 44 s and the maximum interval was 74 s, allowing time for the previous scent to dissipate from the room and prevent olfactory habituation before the next was delivered.

#### 2.2.2 Effort to obtain reward task

A subset of participants completed the effort to obtain reward task (29 non-carriers, 14 asymptomatic carriers, 9 mildly symptomatic carriers, and 27 with bvFTD). Participants were asked if they would like to smell each of the same 7 scents, though in this task they were referred to using colloquial, rather than scientific names to elicit a response based on anticipated valence (vanillin = vanilla, citral = lemon, menthol = mint, isovaleric acid = sweaty feet, pyridine = fish, propionic acid = vinegar, propylene glycol = no smell). Scents were administered in random order. Participants were instructed to respond using a button box with 2 buttons, one labeled “yes” and another labeled “no.” After participants chose to avoid or receive a scent, they were instructed to press the button reflecting their choice as many times as possible to obtain or avoid the smell. A practice trial was administered at the beginning of the task, with the number of button presses on that trial being used to derive an individual threshold for all subsequent trials. Individual thresholds were used for each participant in order to control for individual and disease-related differences in motor ability. The number of button presses during this practice trial was multiplied by 1.1. The resulting value was used as the threshold for all of the following trials, requiring participants to meet or exceed this value in order to obtain the outcome they had chosen. An early iteration of the task defined the threshold as the number of button presses during the practice trial, instead of 10% more than the practice trial result. As the use of a different threshold could have influenced the degree of effort participants exerted across trials, for any analysis involving measures that reflect effort, the 10 participants (3 non-carriers, 1 asymptomatic carrier, 6 bvFTD) tested with the earlier threshold were removed, though they were included in comparisons that solely reflected choice of whether they wished to smell each olfactant or not. The sample for comparisons of effort contained 26 non-carriers, 13 asymptomatic carriers, 9 mildly symptomatic carriers, and 21 with bvFTD.

#### 2.2.3 Odor discrimination task

To confirm the results reflected differences in reward processing and not olfactory ability, an odor discrimination task was administered. Ten pairs of scents were delivered to participants and they were instructed to indicate whether each pair was the same or different. Those who completed this task included 27 non-carriers, 15 asymptomatic carriers, 7 mildly symptomatic carriers, and 27 bvFTD.

### 2.3 Statistics

Our analyses aimed to determine the effect of gene status, disease severity, and any independent or interacting effect of stimulus valence on subjective pleasantness ratings and motivated effort (choice, button press rate and variability, and success rate). To answer these questions, we employed mixed effects models in R, using different model families depending on the class of the outcome variable (R Core Team, 2022; Bates et al., 2015). When the outcome variables of interest did not involve repeated measures, simple ordinal and linear regression models were used in place of mixed effects models (Venables and Ripley, 2002). For analyses involving comparisons of group means, we used ANOVA or a *t*-test. Quantile-quantile plots were used to test for assumptions of normality and Levene’s test to evaluate homogeneity of variance (Kassambara, 2022; Fox and Weisberg, 2019), with a plan to use Kruskal-Wallace if there was a non-normal distribution (Kruskal and Wallis, 1952), or Welch’s ANOVA if there was a violation in the homogeneity of variance assumption (Kassambara, 2022). For all tests that permit nuisance covariates, age and sex were included as covariates with the exception of Welch’s ANOVA, which does not support nuisance covariates. *Post hoc* comparisons for main effects were done with the glht function (Hothorn et al., 2008). The emmeans function (Lenth, 2021) was used for *post hoc* comparisons of interaction terms. For all *post hoc* comparisons, Tukey’s correction was used to account for multiple comparisons.

## 3 Results

Comparisons between groups showed significant differences across measures of disease severity and NPI (Table 1). As expected, patients with bvFTD performed worse in all measures of disease severity and NPI, with the exception of the NPI depression subscale, on which mildly symptomatic carriers scored non-significantly higher.

### 3.1 Reward consumption task

**Figure 1:**
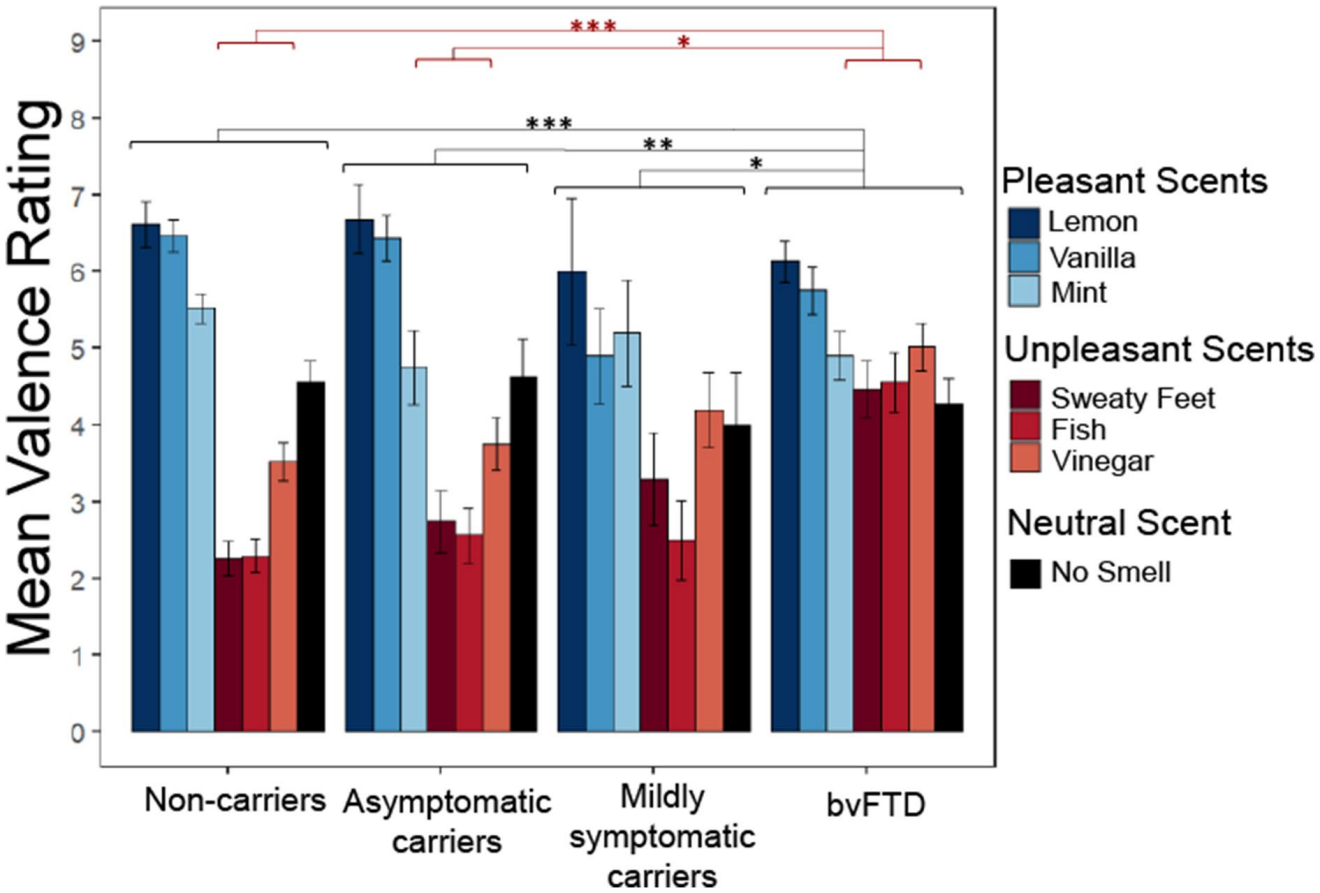
Subjective pleasantness ratings for all scents by group with group*valence (pleasant, unpleasant, neutral) interaction and main effect of group. Pleasantness rated 1–9 with 1 being extremely unpleasant and 9 extremely pleasant. *p < 0.05; **p < 0.01; ***p < 0.001.

We wanted to know whether preclinical carriers of FTD mutations display changes in the perception of pleasantness as previously described in bvFTD, and if so, whether valence perception differs depending on their level of impairment (as a marker of proximity to diagnosis). Figure 1 illustrates how subjective pleasantness ratings shift with increasing disease severity (particularly among mildly symptomatic carriers), including more commonly giving elevated ratings to unpleasant scents.

We employed a linear mixed-effects model with subjective pleasantness ratings as the outcome variable and valence (pleasant, unpleasant, and neutral) and group as predictors. The model yielded a significant main effect of group [χ^2^(9) = 91.003, *p* < 0.001] with *post hoc* comparisons showing patients with bvFTD rated all scents as significantly more pleasant than non-carriers (*z* = 4.763, *p* < 0.001), asymptomatic carriers (*z* = 3.148, *p* = 0.0091) and mildly symptomatic carriers (*z* = 2.583, *p* = 0.047). The significant main effect of valence indicated that, as expected, pleasant, unpleasant, and neutral scents had different pleasantness ratings [χ^2^(8) = 311.33, *p* < 0.001]. There was also a significant group by valence interaction [χ^2^(6) = 87.983, *p* < 0.001], with *post hoc* comparisons showing those with bvFTD rated unpleasant scents more positively than non-carriers [*t*(138) = −4.763, *p* < 0.001] and asymptomatic carriers [*t*(143) = −3.148, *p* = 0.011], but not mildly symptomatic carriers [*t*(156) = −2.583, *p* = 0.052]. There was no significant difference between groups for pleasant or neutral scents. To ensure these findings did not reflect age differences between the groups, an age matched sensitivity analysis was conducted between the 4 groups. The main effect of group remained significant [χ^2^(9) = 41.15, *p* < 0.001] with *post hoc* comparisons again showing bvFTD rated scents as significantly more pleasant than non-carriers (*z* = 3.38, *p* = 0.0041) and asymptomatic carriers (*z* = 2.98, *p* = 0.015). However, mildly symptomatic carriers were no longer significantly different from bvFTD (*z* = 1.99, *p* = 0.19). The main effect of valence also remained significant [χ^2^(8) = 200.05, *p* < 0.001]. The significant group by valence interaction was preserved [χ^2^(6) = 38.97, *p* < 0.001] along with the *post hoc* comparisons showing that patients with bvFTD rated unpleasant scents more positively than non-carriers [*t*(109) = −3.38, *p* = 0.0055] and asymptomatic carriers [*t*(110) = −2.98, *p* = 0.019], but not mildly symptomatic carriers [*t*(112) = −1.99, *p* = 0.2].

**Figure 2:**
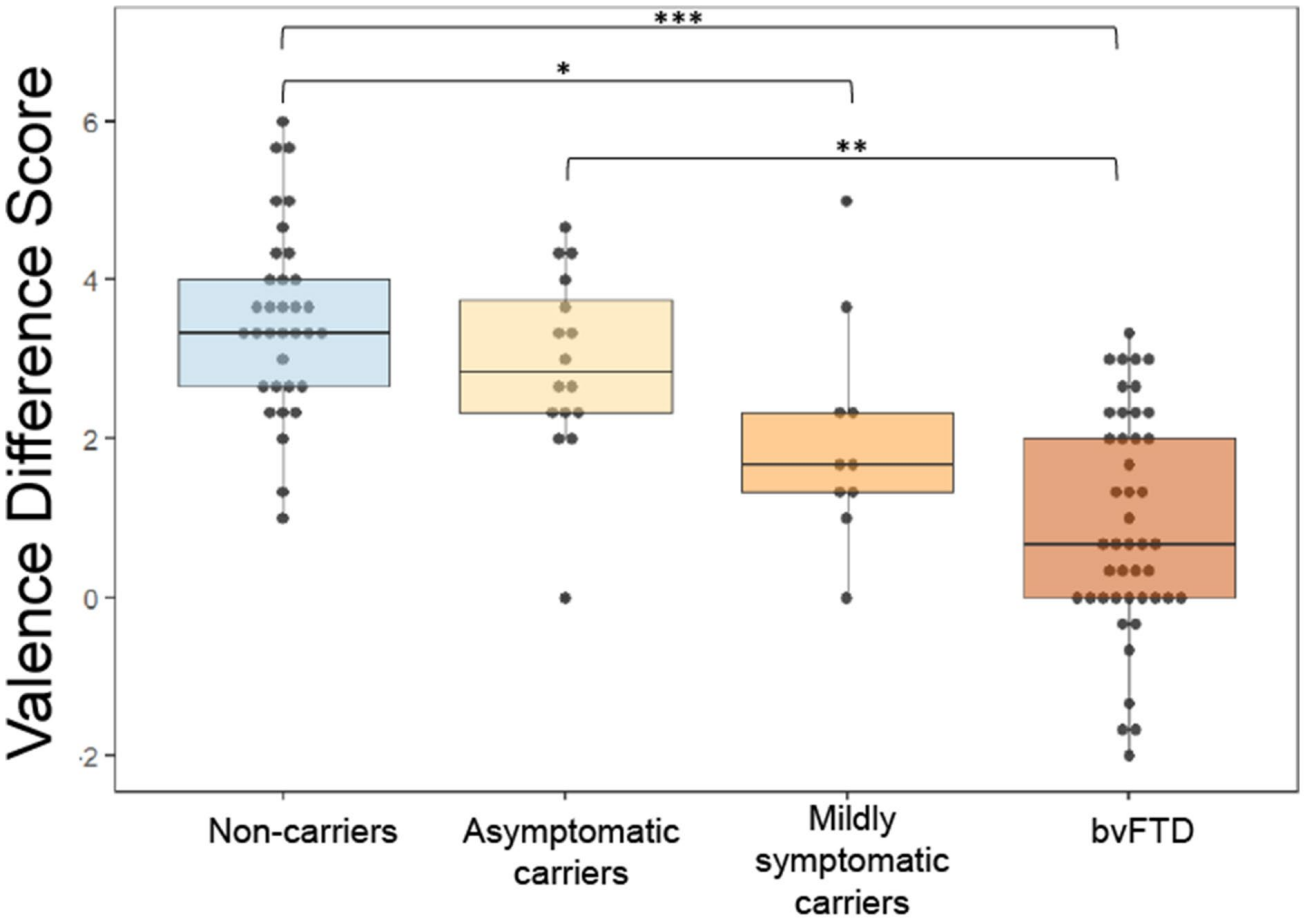
Valence difference scores (mean rating of pleasant scents – mean rating of unpleasant scents) by group. *p < 0.05; **p < 0.01; ***p < 0.001.

We previously found that patients with bvFTD have a reduced difference between subjective ratings of pleasant and unpleasant stimuli (valence difference score), and this has its own anatomic correlates (Perry et al., 2017). We wanted to investigate whether changes in the valence difference score (calculated as mean rating of pleasant scents minus mean rating of unpleasant scents) may occur as an early indication of reward changes in preclinical mutation carriers. Figure 2 shows that with increasing disease severity, the valence difference score approaches the bvFTD finding in preclinical carriers (Figure 2). To compare mean valence difference scores between groups, we conducted an ANCOVA, which yielded a significant difference between group means [*F*(3) = 13.16, *p* < 0.001] with *post hoc* comparisons revealing bvFTD and mildly symptomatic carriers had smaller valence difference scores than non-carriers [mildly symptomatic carriers: t = −2.779, *p* = 0.032; bvFTD: *t* = −6.25, *p* < 0.001]. Patients with bvFTD were also found to have smaller valence difference scores than asymptomatic carriers [*t* = −3.769, *p* = 0.0015] but not mildly symptomatic carriers [*t* = −2.101, *p* = 0.16]. The results were unchanged in an age-matched sensitivity analysis. The mean valence difference scores remained different among groups [*F*(3) = 11.69, *p* < 0.001]. *Post hoc* comparisons showed bvFTD and mildly symptomatic carriers still had significantly smaller valence difference scores than non-carriers [mildly symptomatic carriers: *t* = −2.83, *p* = 0.031; bvFTD: *t* = −5.8, *p* < 0.001] and bvFTD still had significantly smaller valence difference scores than asymptomatic carriers [*t* = −3.54, *p* = 0.004].

To determine if reward changes within each group related to other behavioral measures, mean ratings of unpleasant scents, mean ratings of pleasant scents and valence difference scores were compared with reward-relevant NPI subscale measures, including apathy, depression, eating, euphoria, disinhibition, and aberrant motor behavior. A table with comparisons is included in Supplementary Table S1. Correlations within all 4 groups were considered; however, due to the low frequency of non-zero NPI values in non-carriers and asymptomatic carriers, correlations were not run for these two groups. There was a significant positive correlation between valence difference score and depression in mildly symptomatic carriers (*r* = 0.706, *p* = 0.0106). In mildly symptomatic carriers there were also significant negative correlations between apathy and mean ratings of unpleasant scents (*r* = −0.708, *p* = 0.012) and mean ratings of pleasant scents (*r* = −0.57, *p* = 0.0409).

### 3.2 Effort to obtain reward task

One mildly symptomatic carrier and 2 asymptomatic carriers did not complete the effort task. In the other groups, to ensure there was no bias in who completed this portion of the testing battery, age, gender and MMSE were compared between those that completed the effort to obtain reward task and those that did not within each group. Age and gender did not differ within non-carriers [age: *t*(5.71) = 0.97, *p* = 0.37. gender: χ^2^(1) = 0.68, *p* = 0.41] and bvFTD [age: *t*(36.61) = −0.55, *p* = 0.59. gender: χ^2^(1) = 0.19, *p* = 0.66]. MMSE did not significantly differ within bvFTD [*t*(36.62) = −0.13, *p* = 0.9] but the difference among non-carriers was found to be significant [*t*(26) = 5.41, *p* < 0.001], albeit with a small absolute difference in scores (mean MMSE of 29 among those that completed the effort task and 28 for those who did not).

#### 3.2.1 Choice

**Figure 3:**
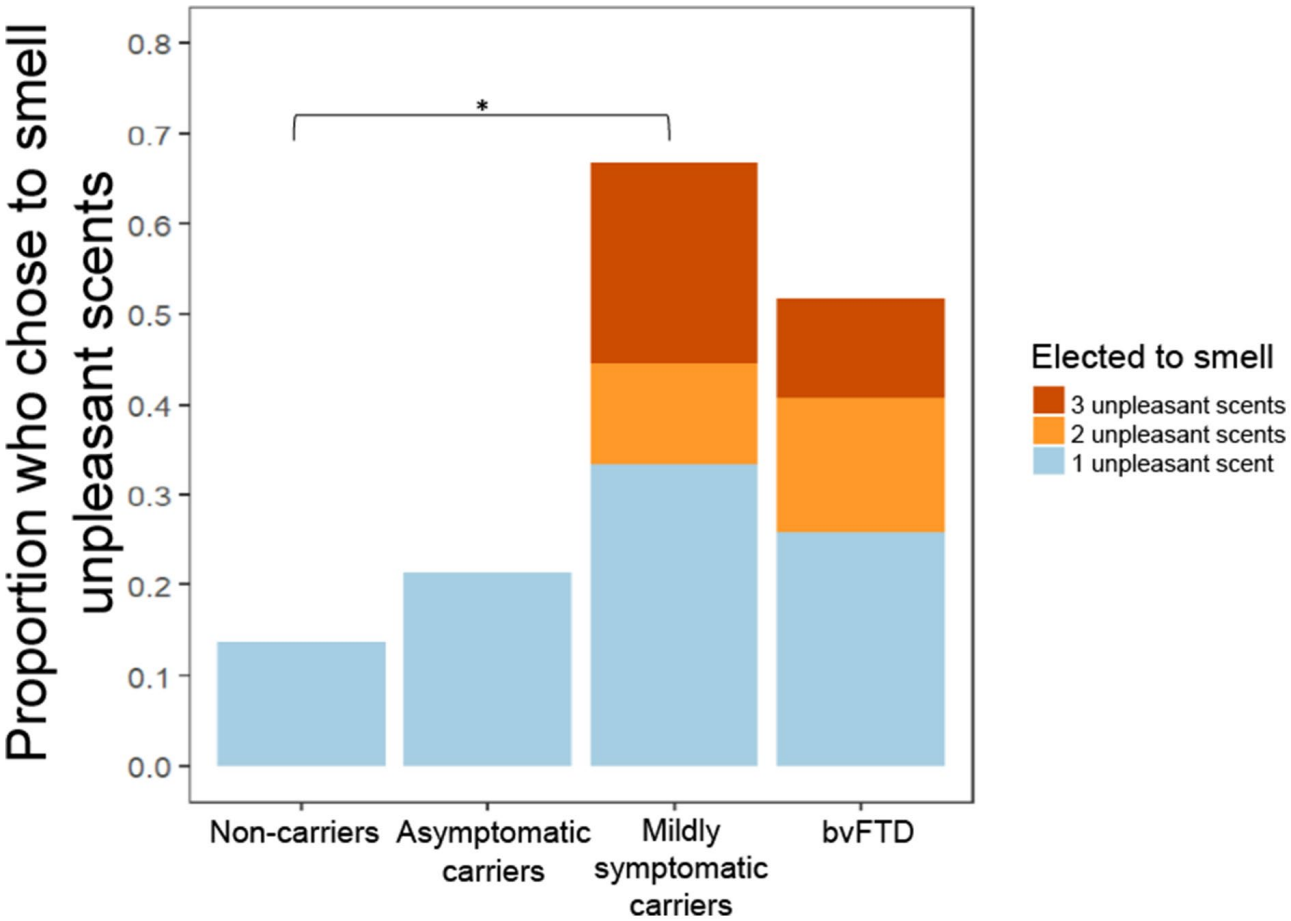
Proportion of participants who chose to smell unpleasant scents by group and number of unpleasant scents chosen. *p < 0.05.

In a previous study, we observed a non-significant trend in which individuals with bvFTD chose to smell unpleasant scents more often and pleasant scents less often than healthy controls (Perry et al., 2017). In the present study, we found that mildly symptomatic carriers had the highest rate of choosing to smell all scents, which was largely driven by their high rate of choosing to smell unpleasant scents (Figure 3). A generalized logistic mixed-effects model was conducted due to the binary outcome variable, the choice to smell or not smell each scent, with group and valence as predictor variables. The model yielded a significant group x valence interaction [χ^2^(7) = 40.044, *p* < 0.001] with *post hoc* comparisons showing mildly symptomatic carriers and the bvFTD group elected to smell unpleasant scents significantly more often than non-carriers (mildly symptomatic carriers: *z* = −3.46, *p* = 0.003; bvFTD: *z* = −2.96, *p* = 0.016). There was also a significant main effect of group [χ^2^(9) = 41.93, *p* < 0.001] with *post hoc* comparisons showing mildly symptomatic carriers and bvFTD chose to smell all scents more often than non-carriers [mildly symptomatic carriers: *z* = 3.46, *p* = 0.0028; bvFTD: *z* = 2.960, *p* = 0.016]. The main effect of valence was also significant [χ^2^(8) = 329.34, *p* < 0.001]. To further explore this finding, we compared the total number of unpleasant scents (0–3) chosen by individuals between groups. Consistent with Figure 3 and the first model, mildly symptomatic carriers chose to smell the highest number of unpleasant scents. Of note, only participants with bvFTD or mildly symptomatic carriers elected to smell more than one of the unpleasant scents (3/9 mildly symptomatic carriers and 7/27 bvFTD), whereas no non-carriers or asymptomatic carriers chose to smell more than one (Figure 3). An ordinal logistic regression model was run with group as the predictor. The model yielded a significant effect of group [χ^2^(3) = 11.5, *p* = 0.0093], with *post hoc* comparisons showing mildly symptomatic carriers elected to smell a greater number of unpleasant scents than non-carriers (*z* = 3.111, *p* = 0.01).

**Figure 4:**
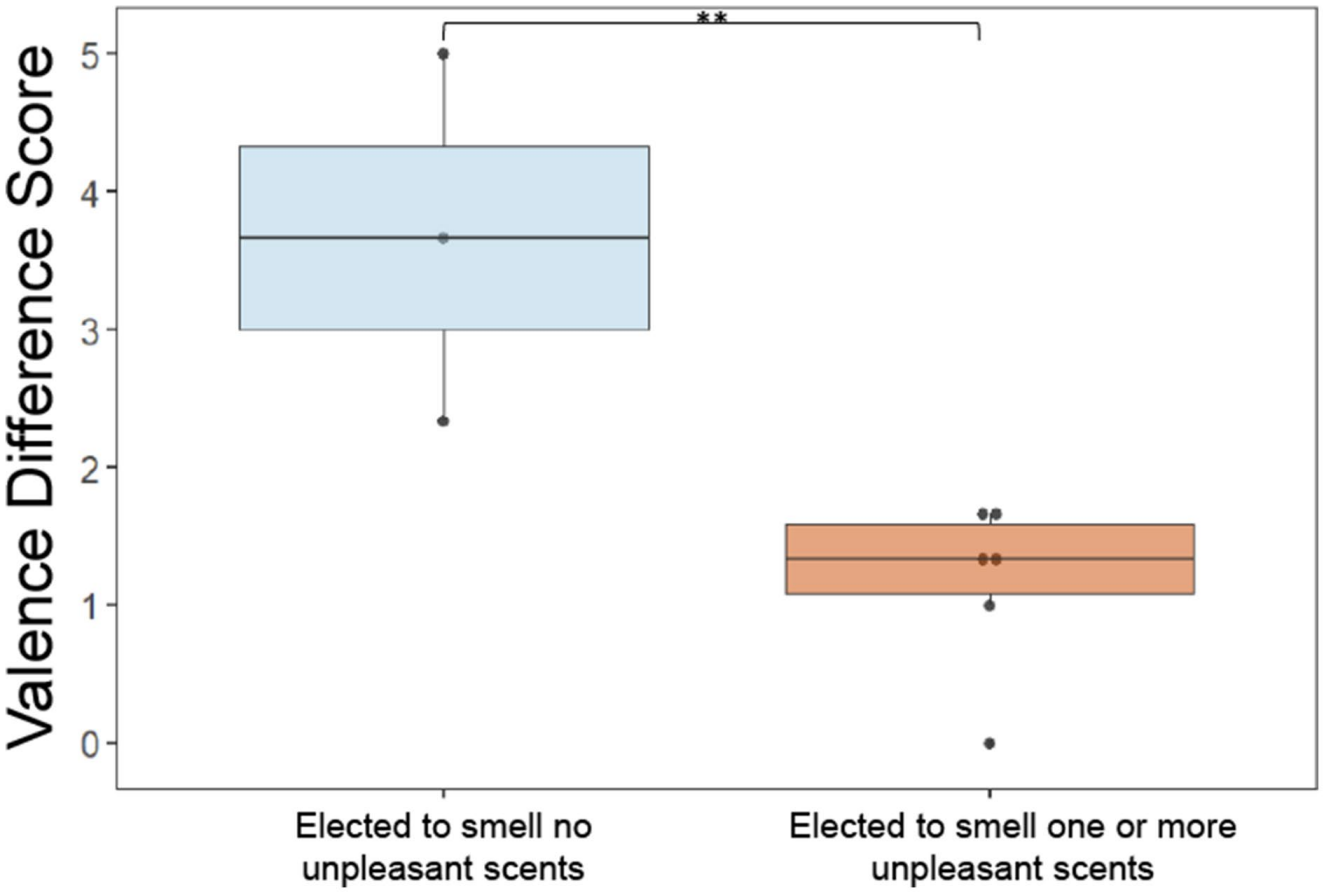
Valence difference score comparison of those in the mildly symptomatic carrier group who chose to smell at least one unpleasant scent and those who chose no unpleasant scents. **p < 0.01.

Next, we used an ordinal logistic regression to assess whether for all participants, electing to smell unpleasant scents was related to subjective pleasantness ratings, with the outcome variable being number of unpleasant scents chosen, group as a nuisance covariate, and the mean ratings of pleasant and unpleasant scents as predictors. Mean rating of unpleasant stimuli was a significant predictor of choosing to smell unpleasant scents [χ^2^(1) = 5.02, *p* = 0.025], but mean subjective pleasantness ratings of positive scents was not [χ^2^(1) = 0.15, *p* = 0.69]. Comparisons focused on mildly symptomatic carriers revealed that those who chose to smell at least one unpleasant scent also had a significantly lower valence difference score than those who elected not to smell any unpleasant scents [*F*(1) = 15.91, *p* = 0.0053] (Figure 4).

#### 3.2.2 Button press and choice

**Figure 5:**
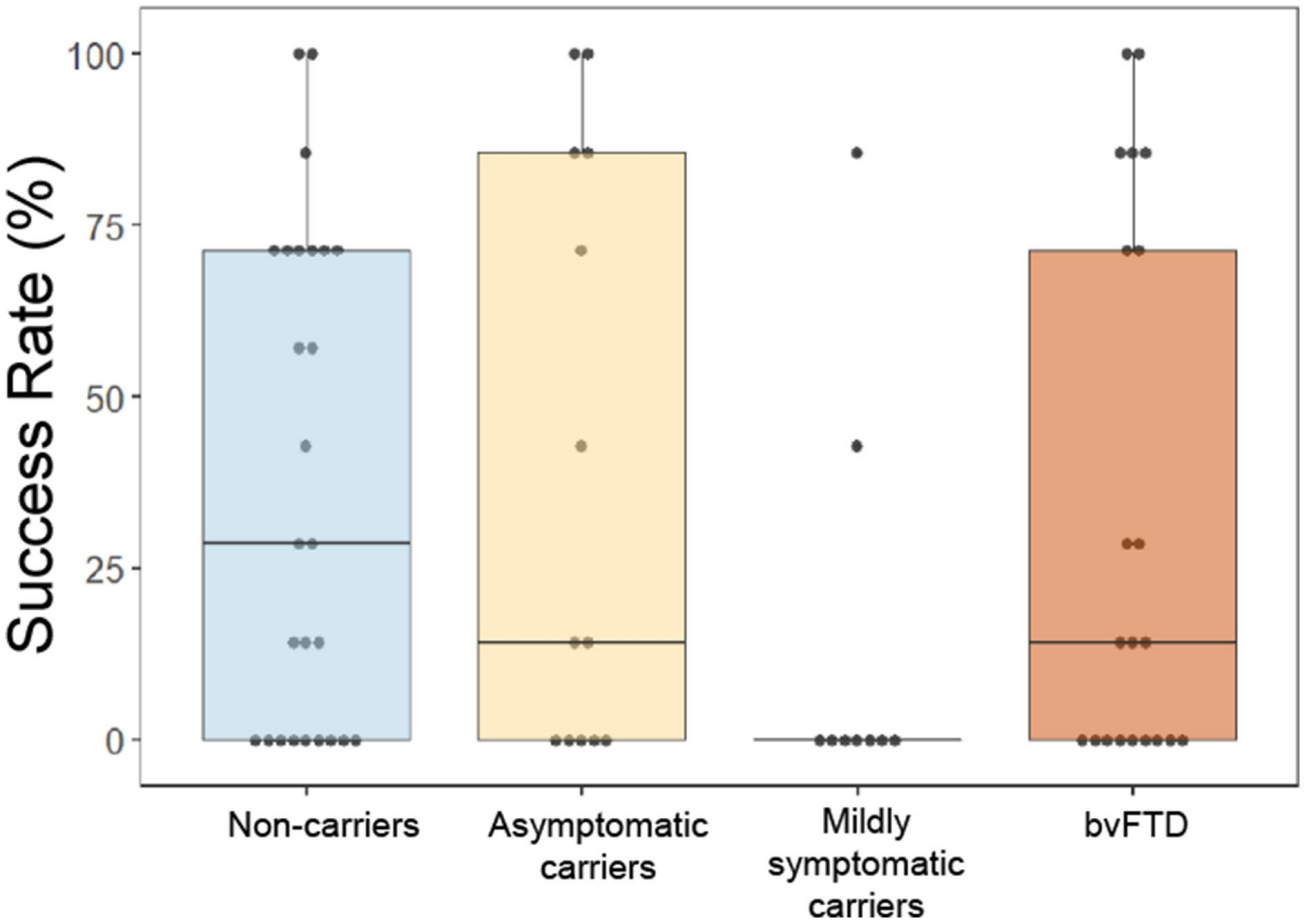
Overall success rate by group at obtaining or avoiding their choices by rapid button pressing on the effort to obtain reward task. *p < 0.05.

Whereas our prior study demonstrated a lower success rate in bvFTD than controls at surpassing the threshold of button presses only when choosing to avoid a scent (Perry et al., 2017), in this cohort we found that mildly symptomatic carriers were the least successful at surpassing threshold of any group, independent of choice and valence, with only 2 participants in this group ever achieving threshold for any of the scents (Figure 5). A generalized logistic mixed model with choice and group as predictors of whether threshold was achieved for each scent revealed a significant main effect of group [χ^2^(6) = 12.69, *p* = 0.048], though *post hoc* comparisons were non-significant (mildly symptomatic carriers and non-carriers: z = −2.047, *p* = 0.17; mildly symptomatic carriers and asymptomatic carriers: *z* = −1.960, *p* = 0.2; all other comparisons: *p* > 0.5). The interaction between choice and group indicated a non-significant trend [χ^2^(3) = 6.41, *p* = 0.093].

**Figure 6:**
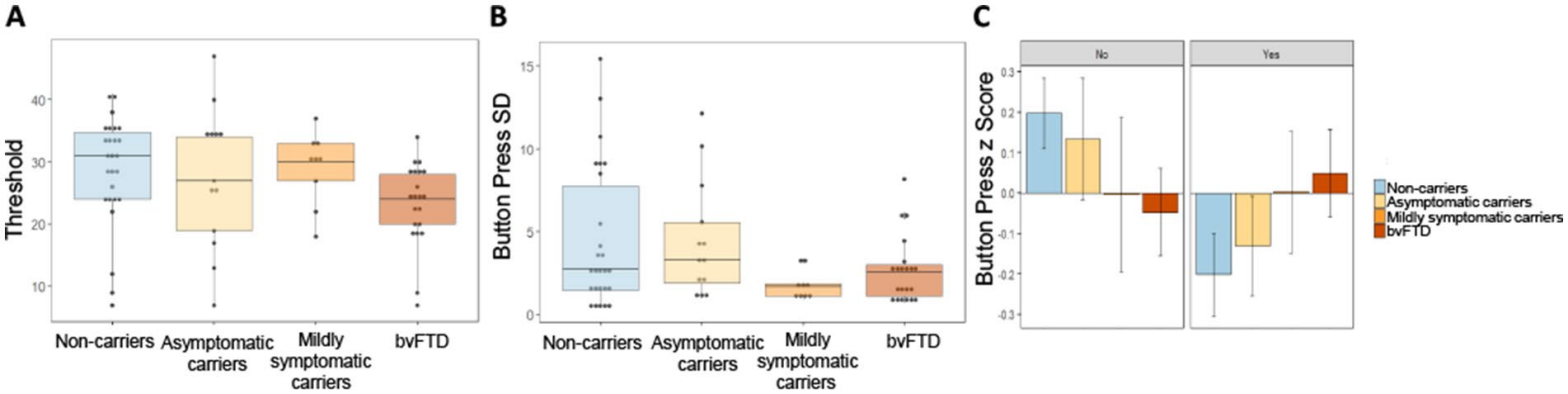
Button press threshold and variability by group. **(A)** Threshold values set during baseline trial by group. **(B)** Intraindividual standard deviation of button presses for all 7 scents by group. **(C)** Intraindividual button press z scores by group and choice of whether to smell or avoid each scent.

To probe the low success rate in mildly symptomatic carriers, we first compared raw threshold values between groups to see if lack of success could be a result of setting higher threshold values during the practice trial. There was little difference in threshold between groups (Figure 6A), and a simple linear model comparing the threshold values yielded a non-significant main effect of group [χ^2^(3) = 212.51, *p* = 0.38]. We then investigated button press rate between groups in a comparison using the button press percentage relative to threshold for each scent in each participant. Due to the outcome variable being a percentage that could take on values outside of 0 and 100%, we used a mixed effects model with a gamma distribution. Neither the main effect of group [χ^2^(6) = 2.073, *p* = 0.91] nor the interaction between choice and group [χ^2^(3) = 1.43, *p* = 0.7] were significant, indicating no difference in button press rate between groups. We then looked at the variance in button presses between groups to explore another potential explanation for differences in successfully obtaining threshold on certain trials, but not others. Intraindividual standard deviation (SD) values were calculated using the button presses for each participant in response to their choice for each of the 7 scents. Figure 6B shows mildly symptomatic carriers had a particularly low SD (Figure 6B). As Levene’s test for homogeneity of variance indicated that variance between the groups was unequal [*F*(3) = 2.89, *p* = 0.042], violating an assumption of ANOVA, we employed Welch’s ANOVA to compare standard deviation between groups. Welch’s ANOVA is not compatible with covariates, thus age and gender were not controlled for; however, to ensure age and gender were not related to intraindividual SD overall or within each group, we ran several correlations, all of which were non-significant (age overall: *r* = −0.16, *p* = 0.19; gender overall: *r* = −0.07, *p* = 0.57; age within group: non-carriers: *r* = −0.149, *p* = 0.47; asymptomatic carriers: *r* = 0.142, *p* = 0.64; mildly symptomatic carriers: *r* = −0.163, *p* = 0.68; bvFTD: *r* = 0.266, *p* = 0.24; gender within group: non-carriers: *r* = −0.28, *p* = 0.17; asymptomatic carriers: *r* = 0.112, *p* = 0.72; mildly symptomatic carriers: *r* = −0.275, *p* = 0.47; bvFTD: *r* = 0.0885, *p* = 0.7). Welch’s ANOVA yielded a significant difference in button press SD between groups [*F*(3) = 4.82, *p* = 0.007] with uncorrected, but not corrected *post hoc* comparisons showing mildly symptomatic carriers had significantly less variability than non-carriers (*p* = 0.039) and a trend toward less variability compared to asymptomatic carriers (*p* = 0.062) and bvFTD (*p* = 0.064). All *post hoc* comparisons were non-significant after correcting for multiple comparisons.

To probe the source of within subject variance we examined intraindividual Z scores for each participant’s button presses split by choice of whether or not to smell a stimulus. Inspection of Figure 6C suggests a trend with increasing disease severity. Unaffected groups (non-carriers and asymptomatic carriers) pressed the button faster when they wished to avoid a scent relative to when they wished to obtain a scent. This trend shifted in advanced disease stages, with mildly symptomatic carriers showing no difference in how quickly they pressed the button whether they wished to obtain or avoid a scent and bvFTD pressing the button faster when they wished to obtain a scent. A linear mixed-effects model with intraindividual z score as the outcome variable and group and choice as predictors, produced a non-significant but trending interaction between choice and group [χ^2^(3) = 6.77, *p* = 0.08]. The main effect of group was non-significant [χ^2^(6) = 6.8, *p* = 0.34].

To determine if effort to obtain reward task measures were related to other behavioral measures within each group, we compared reward-related NPI measures with the number of unpleasant scents participants chose to smell, button press SD and success rate. A table containing all comparisons can be found in Supplementary Table S2. Due to the low frequency of non-zero NPI values in non-carriers and asymptomatic carriers, correlations were not run for these two groups. Mildly symptomatic carriers who chose to smell at least one unpleasant scent had significantly lower depression scores compared with those who chose to avoid all unpleasant scents [two sample *t*-test, *t*(4.14) = 4.27, *p* = 0.012]. There was also a significant negative correlation in mildly symptomatic carriers between NPI depression score and number of unpleasant scents a participant chose to smell (*r* = −0.61, *p* = 0.035). All other comparisons of effort to obtain reward measures and reward relevant NPI subscale measures were non-significant.

### 3.3 Odor discrimination

Twenty-seven non-carriers, 15 asymptomatic carriers, 7 mildly symptomatic carriers, and 27 with bvFTD completed the odor discrimination task. Each participant was given a score between 0 and 1 reflecting the percentage of scent pairs they correctly identified as the same or different. To determine if the mean discrimination scores for each group significantly differed, an ANCOVA was conducted. There was a significant main effect of group [*F*(3) = 3.35, *p* = 0.024] with *post hoc* comparisons showing bvFTD had significantly lower discrimination scores than non-carriers (*t* = −3.1, *p* = 0.014). Due to the difference in discrimination scores, we performed 4 additional analyses to determine whether the ability to discriminate scents influenced subjective pleasantness ratings.

In the first analysis, a linear mixed-effects model was used to compare subjective pleasantness ratings between those with high discrimination scores and those with low discrimination scores. Models were run within each group and included odor valence as a covariate. The mean discrimination score for non-carriers (0.88) was used as the threshold to separate participants by high and low discrimination. Those with discrimination scores ≥0.9 were considered high discrimination and all others were considered low discrimination. 17/27 non-carriers, 8/15 asymptomatic carriers, 4/7 mildly symptomatic carriers and 7/27 bvFTD had high discrimination scores. For none of the 4 groups was there a main effect of discrimination score group on subjective pleasantness ratings [non-carriers: χ^2^(3) = 2.69, *p* = 0.44; asymptomatic carriers: χ^2^(3) = 1.36, *p* = 0.71; mildly symptomatic carriers: χ^2^(3) = 5.25, *p* = 0.15; bvFTD: χ^2^(3) = 2.96, *p* = 0.4] or a discrimination score x valence interaction effect [non-carriers: χ^2^(2) = 1.39, *p* = 0.5; asymptomatic carriers: χ^2^(2) = 4.6, *p* = 0.8; mildly symptomatic carriers: χ^2^(2) = 1.97, *p* = 0.37; bvFTD: χ^2^(2) = 2.26, *p* = 0.32].

In the second analysis, the main valence model was run including only participants with high discrimination scores (≥0.9). The significant main effect of group remained [χ^2^(9) = 21.67, *p* = 0.01] as well as the group x valence interaction [χ^2^(6) = 17.18, *p* = 0.0086]. *Post hoc* comparisons showed mildly symptomatic carriers rated scents as significantly more pleasant than asymptomatic carriers (*z* = 2.62, *p* = 0.043); however, in this much smaller sample, all *post hoc* comparisons for the group x valence interaction were non-significant.

The third analysis was the main valence mixed model with discrimination scores included as a covariate. The significant effect of group [χ^2^(9) = 53.2, *p* < 0.001] as well as group x valence interaction [χ^2^(6) = 52.6, *p* < 0.001] remained. *Post hoc* comparisons of the main effect of group showed patients with bvFTD rated scents as significantly more pleasant than non-carriers (*z* = 2.72, *p* = 0.032). Similarly, *post hoc* comparisons for the interaction term showed bvFTD rated unpleasant scents more positively than non-carriers (*t* = −2.72, *p* = 0.038). There was no significant difference between groups for pleasant or neutral scents.

In the fourth analysis, we conducted the ANCOVA comparing mean valence difference scores between groups, including discrimination score as a covariate. The significant difference between group means was preserved [*F*(3) = 8.19, *p* < 0.001]. *Post hoc* comparisons showed bvFTD had smaller valence difference scores than non-carriers (*t* = −4.503, *p* < 0.001) and asymptomatic carriers (*t* = −3.031, *p* = 0.017); however, the difference between mildly symptomatic carriers and non-carriers was no longer significant (*t* = −2.205, *p* = 0.13).

## 4 Discussion

Characterizing early reward changes in bvFTD may improve diagnostic accuracy and inform symptomatic treatment targets. In a study comparing asymptomatic or early symptomatic carriers of FTD-associated gene mutations with noncarriers and individuals with bvFTD, we found that gene carriers with mild functional changes displayed a similar pattern in their subjective pleasantness ratings for olfactory stimuli to bvFTD, including that they perceived unpleasant stimuli less negatively. Mutation carriers with mild symptoms displayed a blunted perception of the difference in valence between pleasant and unpleasant stimuli. When allowed to choose to smell or avoid different scents, mildly symptomatic carriers elected to smell unpleasant scents more often than their asymptomatic or noncarrier counterparts, with mildly symptomatic carriers even exceeding bvFTD in how often they chose to smell unpleasant scents. This altered approach behavior related to valence perception, with more positive perception of negative stimuli among those who elected to smell negative smells, and in mildly symptomatic carriers, those that elected to smell unpleasant scents having smaller valence difference scores than those that did not. In spite of being more likely to choose to smell stimuli, mildly symptomatic carriers showed a low success rate when required to put forth motivated effort to receive their choice. These findings indicate that alterations in reward perception and approach behavior can be detected early in FTD, prior to meeting diagnostic criteria.

Patients with bvFTD display an altered experience of reward and punishment (Chokesuwattanaskul et al., 2023) and have been shown to prioritize potential gain over loss (Perry et al., 2015; Torralva et al., 2007). This favoring of gain may stem in part from insensitivity to aversive stimuli and outcomes, as reduced sensitivity to aversive things has been observed in bvFTD in a variety of contexts (Perry et al., 2017; Fletcher et al., 2015; Eckart et al., 2012; Grossman et al., 2010). Our study suggests insensitivity to aversive scents emerges early in the disease course and is a key element of altered reward functioning in bvFTD. However, as demonstrated by our valence difference score findings, the shift in perception of valence does not only involve excessively positive perception of negative scents, but an overall restricted valence range, with ratings of pleasant and unpleasant scents beginning to converge in mildly symptomatic carriers. Changes in valence perception in mildly symptomatic carriers may relate to early atrophy or connectivity differences that have been described in regions known to be involved in valence, such as the amygdala, insula, lateral orbital frontal cortex and nucleus accumbens (O’Doherty et al., 2001; Rolls et al., 2003; Becerra and Borsook, 2008; Namburi et al., 2016; Perry et al., 2017).

The finding that mildly symptomatic carriers more often chose to smell unpleasant scents could relate to different potential interpretations, including their blunted perception of negative valence or a bias toward approach behavior. Reward processing includes using prior experience to update stimulus reward value in the form of reward prediction error (Schultz et al., 1997) or change in incentive salience (Berridge, 2012). In our effort to obtain reward task, participants were already familiar with the different scents, having previously completed the reward consumption task, which required them to smell the same seven odors. Thus, choices on the effort task should reflect participants’ recent experience of stimulus pleasantness. The observed correlations between subjective pleasantness ratings and choice to smell unpleasant scents could suggest that this feedback system is intact early in FTD, and the choices of mildly symptomatic patients reflect their subjective experience. Of note, this correlation is not observed in the group with established bvFTD. This may suggest an additional factor weakening the connection between prior experience and action selection in more advanced disease, resulting in greater stochasticity in bvFTD response patterns. Given that both mildly symptomatic carriers and bvFTD show similarly elevated ratings of unpleasant scents, this decoupling in bvFTD may explain mildly symptomatic carriers exceeding bvFTD in their proportional frequency of choosing to smell unpleasant scents. An alternative explanation for why mildly symptomatic carriers chose to smell unpleasant scents, would be that they are biased to pursue or approach, rather than avoid, even in the absence of strong incentive salience. This bias to approach may be a result of underlying atrophy or connectivity changes in regions known to be involved in approach and avoidance behavior, including the amygdala and anterior hippocampus (Miller et al., 2019; Abivardi et al., 2020). Results of the present study suggest some reward alterations, particularly changes in motivated approach behavior evinced by choosing to smell unpleasant scents, may be more pronounced at preclinical timepoints than when functional impairment is greater. These early alterations suggest aberrant valence perception and approach behavior may be candidates for early disease indicators in bvFTD.

Though to our knowledge no previous studies have investigated reward processing in preclinical carriers of FTD mutations, there is evidence of early behavioral changes in other domains. Gene carriers at the CDR 0.5 stage demonstrate significant deficits in socioemotional sensitivity and empathy (Foster et al., 2022; Franklin et al., 2021). Neuropsychiatric symptoms, particularly depression and anxiety, are also observed prior to conversion to full disease and do not always have linear trajectories (Benussi et al., 2021). Other studies, investigating olfactory hedonics in major depression, found that during depressive episodes, patients with greater anhedonia give lower hedonic estimates (Clepce et al., 2010), suggesting that there may be distinctions in reward behavior based on the specific component that is captured by an instrument as depression, whether it is dysphoria, anhedonia, or another cause of withdrawal or decrease in goal-directed behavior. The relationship between depression and reward behavior in this study reflects this complexity; whereas the mildly symptomatic group had non-significantly higher depression scores, both abnormal perception of valence and the high rate of choosing to smell unpleasant stimuli related to low depression scores, suggesting the potential non-linear course of neuropsychiatric symptoms early in the illness, and that the mechanism underlying reward differences may differ between mood disorders and FTD. Similarly, rising apathy would not explain the reward change in presymptomatic carriers; in fact, there was a negative correlation between apathy and subjective pleasantness ratings for negative stimuli, potentially consistent with prior evidence that apathy in bvFTD is primarily driven by effort avoidance rather than changes in reward appetence (Le Bouc et al., 2023).

We found an overall difference between groups in their success rate at surpassing button-press thresholds to receive their choice to smell or avoid various scents. This was primarily driven by strikingly low success in mildly symptomatic carriers. While the groups did not show any difference in overall button press rates or differing thresholds compared to the other groups, follow up analyses suggested this lack of success may relate to a reduction in button press variability, in that mildly symptomatic carriers exert the same effort regardless of scent. This low variability meant fewer outlier, high effort trials that would be more likely to exceed threshold. The analysis of intraindividual z scores for each participant’s button presses was non-significant, but examination suggested a gradual shift from the normal, asymptomatic state that is more motivated to avoid aversive stimuli, toward the bvFTD phenotype in which there is greater effort to obtain pleasant stimuli and avoid aversive ones. This is consistent with our previous study, in which we found that, compared to controls, patients with bvFTD were more motivated to obtain scents they found pleasant and less motivated to avoid scents they found aversive (Perry et al., 2017). At the mildly symptomatic stage, the motivation for approach and avoidance were roughly equal, similar to their subjective pleasantness ratings, which also reflected little difference between positive and negative.

There are several limitations to the study. The first is the reliance on participants sense of smell. Other studies suggest patients with bvFTD have difficulty identifying odors but do not have issue with odor discrimination (Silva et al., 2019). As in our prior study, participants with bvFTD were not as successful as non-carriers at discriminating odors. In spite of analyses run to account for the effect of odor discrimination, it is possible olfactory acuity has some influence on the findings. We ran our main valence model including only participants considered to have high discrimination scores and though this yielded a significant main effect of group, *post hoc* comparisons were non-significant. Moreover, the main valence model and the valence difference score ANCOVA were both run with discrimination score as a covariate and although the differences between bvFTD and the least affected groups were preserved, pairwisecomparisons involving mildly symptomatic carriers lost significance, potentially related to the smaller sample size as there were similar effect sizes. An additional limitation was our small sample size, particularly in the asymptomatic and mildly symptomatic carrier groups, which prevented the analysis of these groups broken down by gene mutation. Thus, we were not able to identify potential genetic heterogeneity. Though *C9orf72, GRN and MAPT* mutations most often result in bvFTD, they occasionally result in other neurodegenerative syndromes including primary progressive aphasia, amyotrophic lateral sclerosis, corticobasal syndrome, progressive supranuclear palsy, and parkinsonism (Le Ber et al., 2008; Kelley et al., 2009; Convery et al., 2019). Therefore, we cannot know with certainty what syndrome symptomatic gene carriers might develop, or if this syndromic heterogeneity would lead to variable reward processing changes. Furthermore, there is evidence that patients with bvFTD exhibit a global reduction in variance of self-reported response patterns which could potentially contribute to our observation of an overall restricted valence range in these patients (Williams et al., 2023). A final limitation of the study is the use of CDR as a surrogate measure for proximity to a bvFTD diagnosis. Receiving a CDR score of 0.5 from a blinded rater is no guarantee of impending conversion to the full disease state.

Given the known alterations to reward functioning in bvFTD, investigating early reward changes in preclinical carriers of FTD mutations has the potential to contribute to our understanding of how this disease progresses in early stages and aid in early and accurate detection. In this study, we found reward alterations can be detected prior to conversion to the full disease state via subjective pleasantness ratings and motivated approach behavior. Future studies could test whether this finding holds using different sensory modalities and in other punishing or aversive paradigms. In addition, investigations of the neural underpinnings of these early reward shifts will help to pinpoint the first reward related regions affected in bvFTD and contribute to understanding the function of these structures. Symptomatic interventions targeting reward-related behavioral symptoms may also be more effective early in the disease course than treatments given in later stages.

## Data Availability

The code and E-Prime files used to administer the reward task are deposited in the OSF repository, DOI 10.17605/OSF.IO/9ZF5S. Public archiving of the anonymized data is not permitted under the study’s IRB approval due to the sensitive nature of patient data; however, data are available upon request by submitting a UCSF MAC Resource Request form: https://memory.ucsf.edu/research-trials/professional/open-science. Following a UCSF-regulated procedure, access will be granted in line with the ethical guidelines on the reuse of sensitive data, including submission of a Material Transfer Agreement, available at: https://icd.ucsf.edu/materialdata-transfer-agreements.
