# Supplementary material for: Reward processing deficits arise early in familial frontotemporal dementia
Source: Front Neurosci. 2024 Nov 6;18:1491972. doi: 10.3389/fnins.2024.1491972 (PMC11576384; doi:10.3389/fnins.2024.1491972)
Supplement: Supplementary file 1 [file Table_1.docx]

Supplementary Material

# Supplementary Figures and Tables

## Supplementary Tables

**Supplementary Table 1.** Results displayed as correlation coefficient (*p*-value). Comparisons of reward consumption task measures with NPI scores within each group. Kendall’s Tau was used to determine the relationship between NPI and reward measures due to the non-parametric nature of the sample. Correlations only run for comparisons with more than 2 non-zero NPI scores.

|  | Mean rating of unpleasant scents | | Mean rating of pleasant scents | | Valence difference score | |
| --- | --- | --- | --- | --- | --- | --- |
| Group | Mildly symptomatic carriers | bvFTD | Mildly symptomatic carriers | bvFTD | Mildly symptomatic carriers | bvFTD |
| NPI apathy | -.708 (.012) * | .058 (.64) | -.57 (.0409) * | .087 (.49) | -.27 (.33) | .071 (.57) |
| NPI depression | -.15 (.59) | -.14 (.27) | .44 (.11) | -.018 (.89) | .706 (.0106) * | .12 (.34) |
| NPI eating | .24 (.41) | -.13 (.29) | .1 (.73) | -.073 (.55) | 0 (1) | .057 (.64) |
| NPI motor | <2 non-zero NPI values | -.019 (.88) | <2 non-zero NPI values | -.068 (.58) | <2 non-zero NPI values | .018 (.88) |
| NPI disinhibition | -.38 (.2) | -.18 (.14) | -.17 (.56) | -.12 (.32) | -.27 (.35) | .15 (.207) |
| NPI euphoria | <2 non-zero NPI values | -.29 (.017) * | <2 non-zero NPI values | -.12 (.32) | <2 non-zero NPI values | .36 (.0029) ** |

**Supplementary Table 2.** Results displayed as correlation coefficient (*p*-value). Comparisons of effort to obtain reward task measures with NPI scores within each group. Kendall’s Tau was used to determine the relationship between NPI and reward measures due to the non-parametric nature of the sample. Correlations only run for comparisons with more than 2 non-zero NPI scores.

|  | Number of unpleasant scents chosen | | Button Press SD | | Success Rate | |
| --- | --- | --- | --- | --- | --- | --- |
| Group | Mildly symptomatic carriers | bvFTD | Mildly symptomatic carriers | bvFTD | Mildly symptomatic carriers | bvFTD |
| NPI apathy | -.3002 (.31) | .062 (.74) | -.0308 (.91) | .093 (.62) | .42 (.18) | .14 (.49) |
| NPI depression | -.61 (.035) * | -.065 (.72) | .09 (.75) | -.087 (.64) | .18 (.56) | -.045 (.83) |
| NPI eating | 0 (1) | -.18 (.32) | .27 (.35) | -.23 (.2) | .15 (.63) | -.0804 (.68) |
| NPI motor | <2 non-zero NPI values | -.016 (.93) | <2 non-zero NPI values | -.15 (.41) | <2 non-zero NPI values | -.11 (.56) |
| NPI disinhibition | -.26 (.4) | -.12 (.5002) | .206 (.48) | -.14 (.46) | .62 (.055) | .37 (.059) |
| NPI euphoria | <2 non-zero NPI values | -.25 (.15) | <2 non-zero NPI values | -.27 (.15) | <2 non-zero NPI values | .082 (.68) |
